# *In Silico* Infection Analysis (iSFA) Identified Coronavirus Infection and Potential Transmission Risk in Mammals

**DOI:** 10.3389/fmolb.2022.831876

**Published:** 2022-02-08

**Authors:** Yanyan Zou, Xiaojian Cao, Bing Yang, Lulu Deng, Yangyang Xu, Shuang Dong, Wentao Li, Chengchao Wu, Gang Cao

**Affiliations:** ^1^ State Key Laboratory of Agricultural Microbiology, Huazhong Agricultural University, Wuhan, China; ^2^ College of Informatics, Huazhong Agricultural University, Wuhan, China; ^3^ College of Veterinary Medicine, Huazhong Agricultural University, Wuhan, China; ^4^ Department of Medical Oncology, Hubei Cancer Hospital, Huazhong University of Science and Technology, Wuhan, China; ^5^ Bio-Medical Center, Huazhong Agricultural University, Wuhan, China

**Keywords:** coronaviruses, in silico infection analysis, in silico docking, data mining, COVID-19

## Abstract

Coronaviruses are a great source of threat to public health which could infect various species and cause diverse diseases. However, the epidemic’s spreading among different species remains elusive. This study proposed an *in silico* infection analysis (iSFA) system that includes pathogen genome or transcript mining in transcriptome data of the potential host and performed a comprehensive analysis about the infection of 38 coronaviruses in wild animals, based on 2,257 transcriptome datasets from 89 mammals’ lung and intestine, and revealed multiple potential coronavirus infections including porcine epidemic diarrhea virus (PEDV) infection in *Equus burchellii*. Then, through our transmission network analysis, potential intermediate hosts of five coronaviruses were identified. Notably, iSFA results suggested that the expression of coronavirus receptor genes tended to be downregulated after infection by another virus. Finally, binding affinity and interactive interface analysis of S1 protein and ACE2 from different species demonstrated the potential inter-species transmission barrier and cross-species transmission of SARS-CoV-2. Meanwhile, the iSFA system developed in this study could be further applied to conduct the source tracing and host prediction of other pathogen-induced diseases, thus contributing to the epidemic prevention and control.

## Introduction

Coronaviruses are a great source of threat to humans which causes diverse diseases ranging from asymptomatic to severe respiratory defection (Kahn and McIntosh, 2005). Generally, coronaviruses could be divided into four subtypes, including alpha-coronavirus, beta-coronavirus, delta-coronavirus, and gamma-coronavirus. Some of the known beta-coronavirus such as SARS-CoV, MERS-CoV, and recently emerged SARS-CoV-2 can lead to fatal repository health crisis in humans. Zoonotic transmission routes of coronaviruses usually include natural reservoir and several intermediate hosts, and end with infection of various human cells, causing distinct pathogenic effects in different hosts (Graham and Baric, 2010). The traditional epidemiological research methods which include statistics on the prevalence of large group, retrospective analysis have played an important role in clarifying the epidemic pattern, formulating prevention and control countermeasures of emerging infectious diseases (Cho, 2020; Qiu et al., 2020). However, its traceability analysis is often restricted by tedious and limited sample collection and testing work which may expend much manpower and financial resources (Chen et al., 2020b; Hao et al., 2020).

The invasion of zoonotic coronaviruses to human cells is mediated by spike protein binding to various cell surface receptors, including ACE2, ANPEP, and DPP4. During the infection of SARS-CoV-2, the spike protein of SARS-CoV-2 binds to the ACE2 receptor of human cells, leading to virus invasion via endocytosis. Afterward, the virus–ACE2 complex is delivered into acidified phagolysosome. Then viral RNA is released, which consequently initiates viral replication and the assembly of the progeny viruses (Simmons et al., 2005). The risk level of coronavirus infection is associated with the binding affinity between the virus spike protein and host cell receptors. ANPEP is usually regarded as a receptor of a mild human coronavirus HCoV-229E, while it has been also suggested as one of the receptors for SARS-CoV (Yu et al., 2003). DPP4 has been reported to possess a highly conserved domain among Camelidae, primates, and Leporidae which is highly affinitive to MERS-CoV spike protein, making it one of the major mediators to MERS (Widagdo et al., 2019). However, the interaction of the diverse receptors and the ligands in different coronaviruses and the subsequent cross-species transmission remains elusive.

One of the most effective measures to control the coronavirus epidemic is cutting off the intermediate transmission route. Thus, it is urged to identify the intermediate host of the corresponding virus and the coronavirus infection among different species. Unfortunately, this effort confronts huge challenges due to the requirement of comprehensive screening. The intermediate host for SARS-CoV-2 remains undetermined more than a year after the burst of the epidemic. This study aims to explore the transmission route of coronaviruses in mammals and SARS-CoV-2 potential intermediate hosts. For this purpose, we nominated this integrated computational approach called *in silico* infection analysis to screen coronavirus infection in 89 non-human mammals and systematically analyzed the expression level of coronavirus receptors and binding affinity with the spike protein of coronaviruses.

## Results

### *In Silico* Infection Analysis Identified Coronavirus Infection in Mammals

**FIGURE 1:**
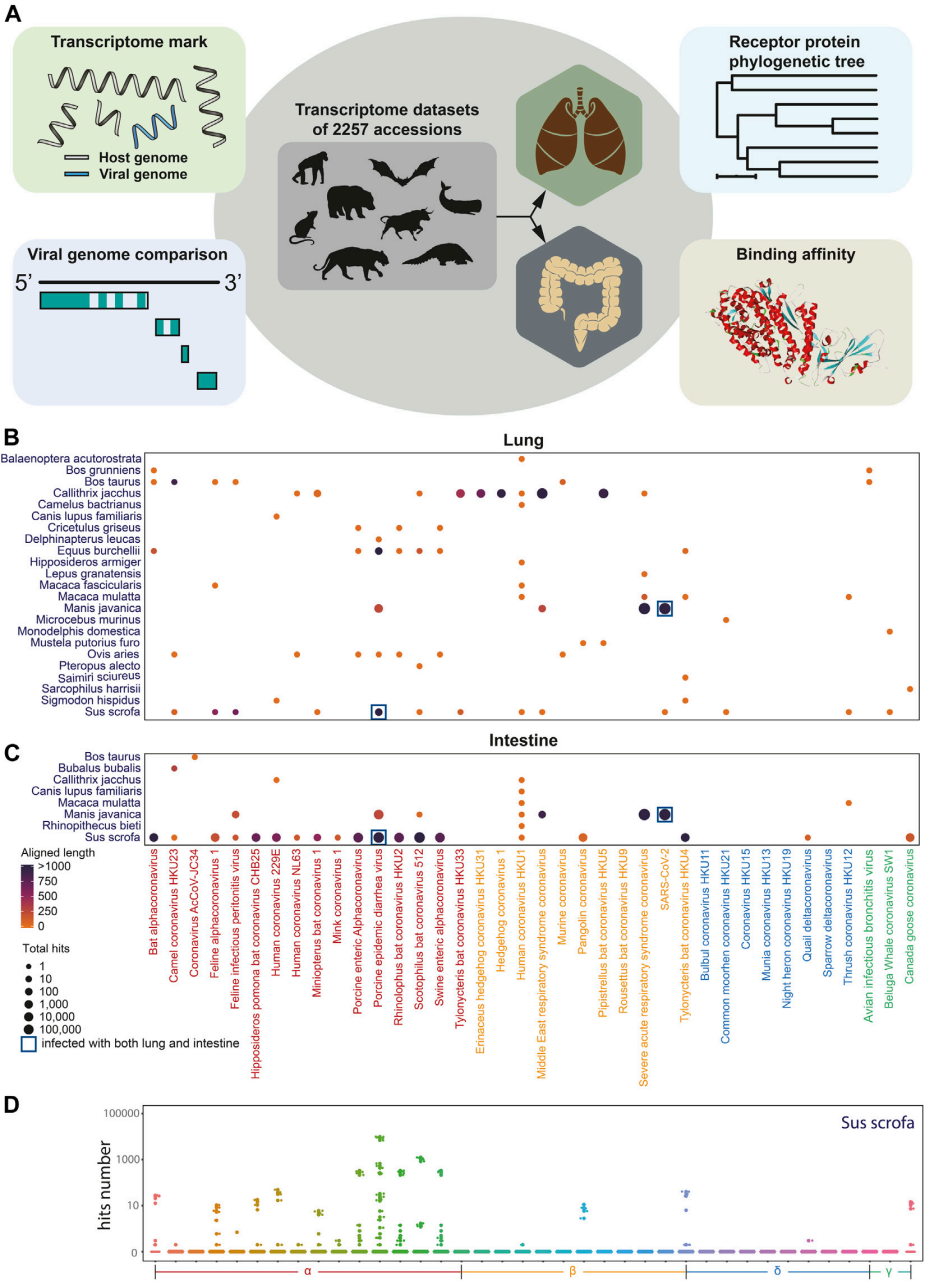
Coronavirus infection landscape. **(A)**. Diagram of coronavirus *in silico* infection analysis in wild animals including coronavirus genome screening, genome comparison and phylogenetic tree analysis, viral receptor genes expression analysis, and viral ligand–receptor binding affinity analysis. **(B)** Coronavirus infection landscape of mammals’ lung and intestine. The size of dots represented the total hits that aligned to the coronaviral genome and the color of dots represented the total aligned length. Navy blue square frame demonstrates the species infected with coronaviruses in both the lung and the intestine. **(C,D)** Box plot of aligned hits number with *Sus scrofa* as hosts.

To explore the coronavirus infection in mammals, we performed *in silico* coronaviral genome screening in mammalian transcriptome datasets, except for widely studied *Homo sapiens*, *Mus musculus*, and *Rattus norvegicus* including lungs and intestines (Figure 1A). In this *in silico* infection analysis, the sequence of the genomic fragment of 38 coronaviruses was screened from 388 intestine datasets and 1,869 lung datasets of 89 mammal species from the NCBI Sequence Read Archive (SRA). Coronavirus genome fragments could not be detected in most of the selected transcriptomic datasets (Supplementary Figures S1A,B). While some fragments of the coronavirus genome can be observed in certain species (Figures 1B,C) including porcine epidemic diarrhea virus (PEDV) identification in both the lung and the intestine of *Sus scrofa* (Figure 1D) and the lung of *Equus burchellii*. Importantly, numerous fragments of SARS-CoV-2–like virus existed in both the lung and intestine tissue of *Manis javanica*, which has been proposed as an intermediate host during the spread of SARS-CoV-2 (Lam et al., 2020). The virus fragments identified in *Equus burchellii* and *Manis javanica* were further analyzed for the exploration of the specific infection strain*.*

### Potential Coronavirus Infection and Transmission Risk Analysis Among Different Mammals

**FIGURE 2:**
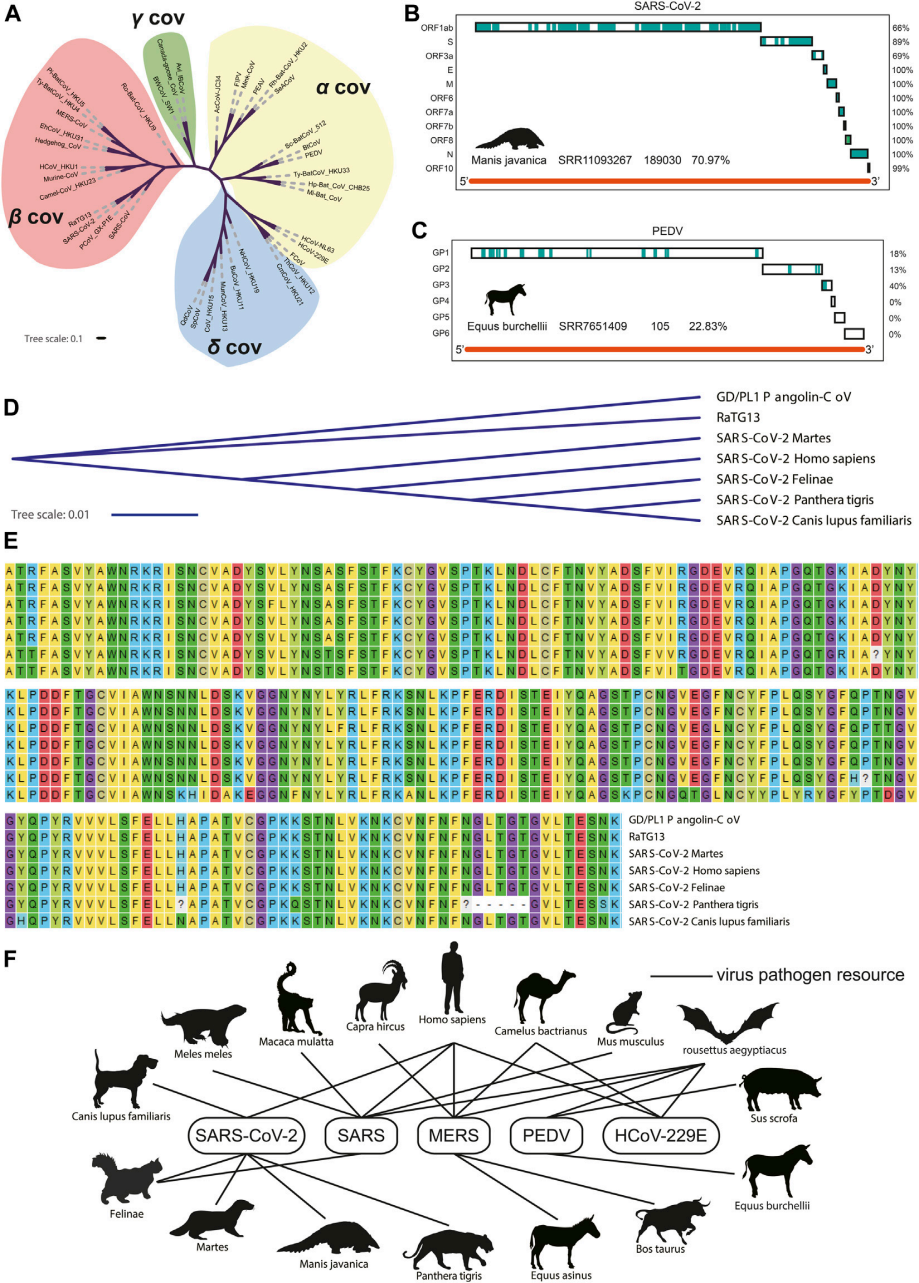
Phylogenetic tree of 38 coronaviruses and potential transmission network. **(A)**. Phylogenetic tree of 38 coronaviruses. **(B)** Coverage of fragments isolated from *Manis javanica* where the blue blocks mean consistent with the SARS-CoV-2 genome **(C)** Coverage of fragments isolated from *Equus burchellii* where the blue blocks mean consistent with PEDV genome. **(D)** Phylogenetic tree of S1 protein. **(E)** Amino acid sequence alignment results. **(F)** Potential transmission network of five coronaviruses between different species.

All the nucleotide sequences of spike (S/GP2) protein were collected from 38 coronaviruses to construct the phylogenetic tree (Figure 2A). As shown in Figures 2B,C, the coronaviruses genome fragments were collected from lung and intestine transcriptome datasets of *Manis javanica* and *Equus burchellii*. The coronaviruses collected from *Manis javanica* and *Equus burchellii* showed the highest genome coverage rate, and most unique viral fragments can be mapped to the SARS-CoV-2 and PEDV genome, respectively. The result of identifying SARS-CoV-2 in *Manis javanica* is consistent with studies (Lam et al., 2020) reported earlier. As for the control group, we also collected the viral fragments isolated from the infection experiments of known strains, which exhibited a high degree of compliance (Supplementary Figures S2A,B). The S1 nucleotide and amino acid sequence of SARS-CoV-2 isolated from *Manis javanica*, *Chiroptera*, *Martes*, Felinae, *Panthera tigris*, *Canis lupus familiaris*, and *Homo sapiens* were further collected and subjected to nucleotide sequence phylogenetic tree and protein sequence alignment analysis, respectively (Figures 2D,E). These data demonstrated that GD/PL1 pangolin-CoV (Lam et al., 2020) and RaTG13 shared the highest nucleotide sequence similarity among species. Finally, we selected five coronaviruses including SARS-CoV-2, SARS-CoV, MERS-CoV, PEDV, and HCoV-229E for the infection network construction between 16 different mammals. Based on our transcriptome data screening and previously reported coronavirus host studies (Stadler et al., 2003; van der Hoek et al., 2004; Manzanillo et al., 2013; Mackay and Arden, 2015; Rahimi and Tehranchinia, 2020). Figure 2F demonstrated that coronaviruses have interlaced the transmission network and are widely spread across species. *Chiroptera* seems to be a central reservoir host of coronaviruses as most coronaviruses can use it as a host (Maxmen, 2017).

### Expression Patterns of Coronavirus Receptors Among Species

**FIGURE 3:**
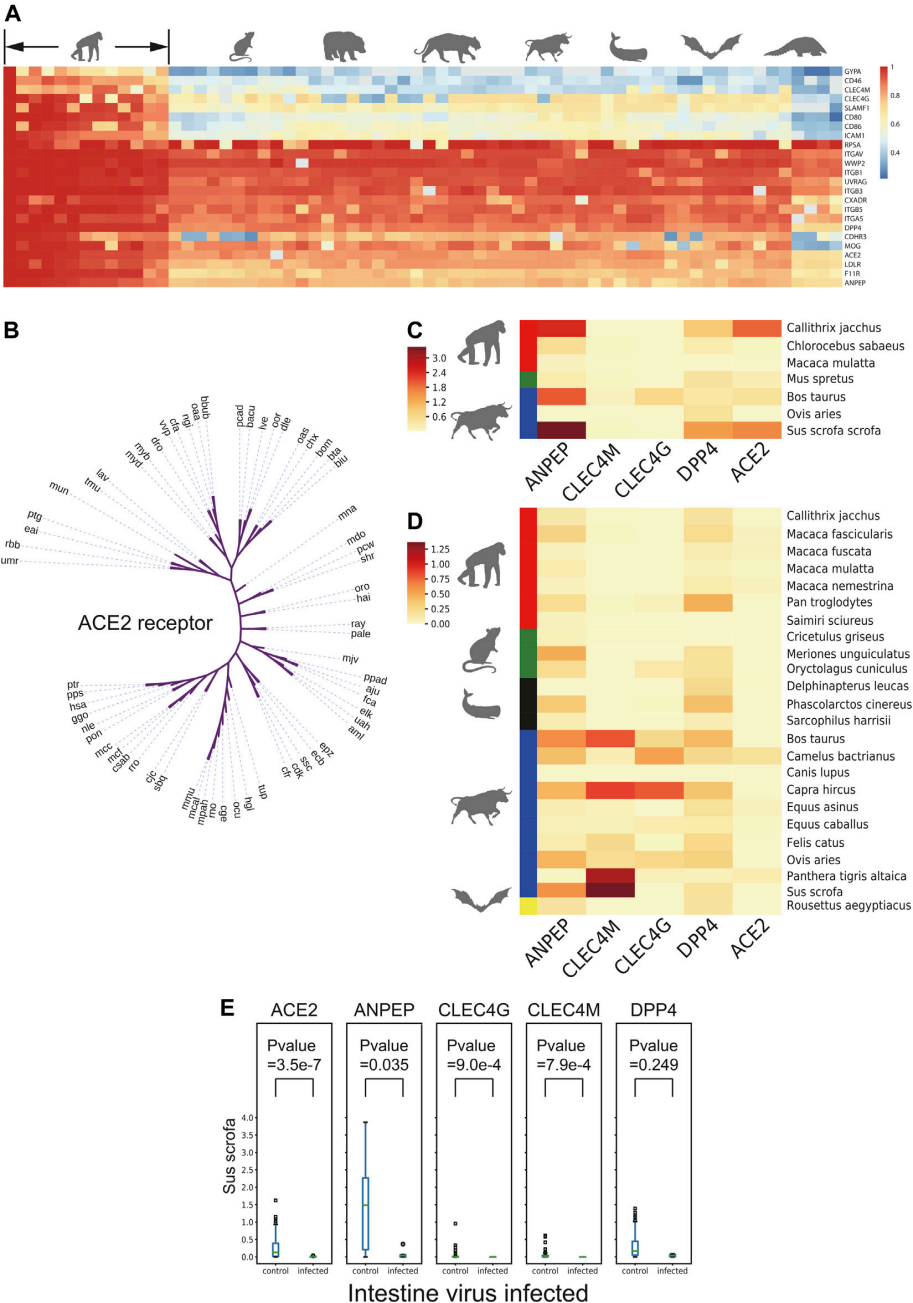
Receptor conservation and expression level analysis. **(A)** Heatmap of sequence similarity of 24 respiratory receptors across 65 species. **(B)** Cladogram of ACE2 across 65 species. **(C)** and **(D)** Heatmap of natural logarithm of mean expression of five viral receptor genes expression in intestine and lung cross-species. **(E)** Box plot of natural logarithm of five receptor genes expression level between PEDV infected and non-infected group in intestine of *Sus scrofa*. The number in brackets means infected/non-infected sample size.

To further evaluate the coronavirus transmission risk across species, the corresponding protein sequences of 24 putative respiratory virus receptors from 65 species (Supplementary Table S2), including *Primates*, *Rodentia*, *Carnivora*, *Artiodactyla*, *Perissodactyla*, *Cetacea*, *Chiroptera*, and *Pholidota* were collected and aligned (Figure 3A). Coronavirus receptors of non-human primates showed high sequence similarities with human receptors, which implied the similar binding affinity to coronavirus ligand. For example, gorilla and monkey, the nearest neighbors in the cladogram (Figure 3B), may be at similar potential risks of SARS-CoV-2 infection as they shared similar ACE2 receptor protein sequences, while some receptors of the rest species demonstrated distinct levels of protein sequence divergence, implying that the infection risk might differ among these species.

As the expression level of virus receptors may reflect the infection probability to some extent of certain viruses, the expression level of virus receptors was analyzed across the species from 1,869 lung transcriptomic datasets and 388 intestine transcriptomic datasets of different mammals (except for commonly studied *Homo sapiens*, *Mus musculus*, and *Rattus norvegicus*). After quality control steps, we kept the sequences of 26 mammalian species for further expression analysis. A total of 24 receptors including ACE2, a receptor of HCoV-NL63, SARS-CoV, and SARS-CoV-2; ANPEP, a receptor of several alpha-coronaviruses and delta-coronaviruses; CLEC4G and CLEC4M, putative receptors of SARS-CoV; and DPP4, a receptor of MERS-CoV and HKU4, were subjected to this expression analysis (MacLachlan and Dubovi, 2011) (Supplementary Figures S3A,B). As shown in Figures 3C,D, the receptor ANPEP presented relatively higher expression in the intestine, while the expression level of CLEC4M remained low in all tested species. Importantly, those five receptors were observed to be less expressed in *Macaca mulatta*. ANPEP has a similar expression pattern in the lung as in the intestine, while DPP4 generally showed higher expression than ACE2 across the species. As *Artiodactyla* mammals, the expression level of CLEC4M and CLEC4G were higher than other species.

Next, to study whether a virus infection would alter receptor gene expression of other viruses, the transcriptome data from *Sus scrofa* with or without PEDV infection in the intestine and lung transcriptome data with or without porcine reproductive and respiratory syndrome virus (PRRSV) infection were taken into further study. The expression level of different virus receptor genes including ACE2, ANPEP, CLEC4G, CLEC4M, and DPP4 were analyzed. Interestingly, we observed that after infection, CLEC4G, the putative receptor gene of SARS, was significantly downregulated in the lung of the PRRSV-infected pigs compared to the non-infected pigs (Supplementary Figure S3C). Notably, more receptor-encoding genes including ACE2, ANPEP, CLEC4G, and CLEC4M were significantly downregulated upon PEDV infection (Figure 3E) in the intestine. These data suggested that infection of a certain virus may suppress the expression of other virus receptors to avoid invasion of other viruses, which could maintain long-term persistence.

### Binding Affinity Analysis of SARS-CoV-2 S1-RBD With ACE2 From Different Species

The protein sequences of ACE2 from 99 species were downloaded from the NCBI, then the homolog sequence was extracted for the structure simulation with the SARS-CoV S1-human ACE2 complex (PDB: 6acg) and the SARS-CoV-2 S1-human ACE2 complex (PDB: 7DF4) as the template with MODELLER. The strategy of *in silico* docking is shown in Supplementary Figure S4A.

**FIGURE 4:**
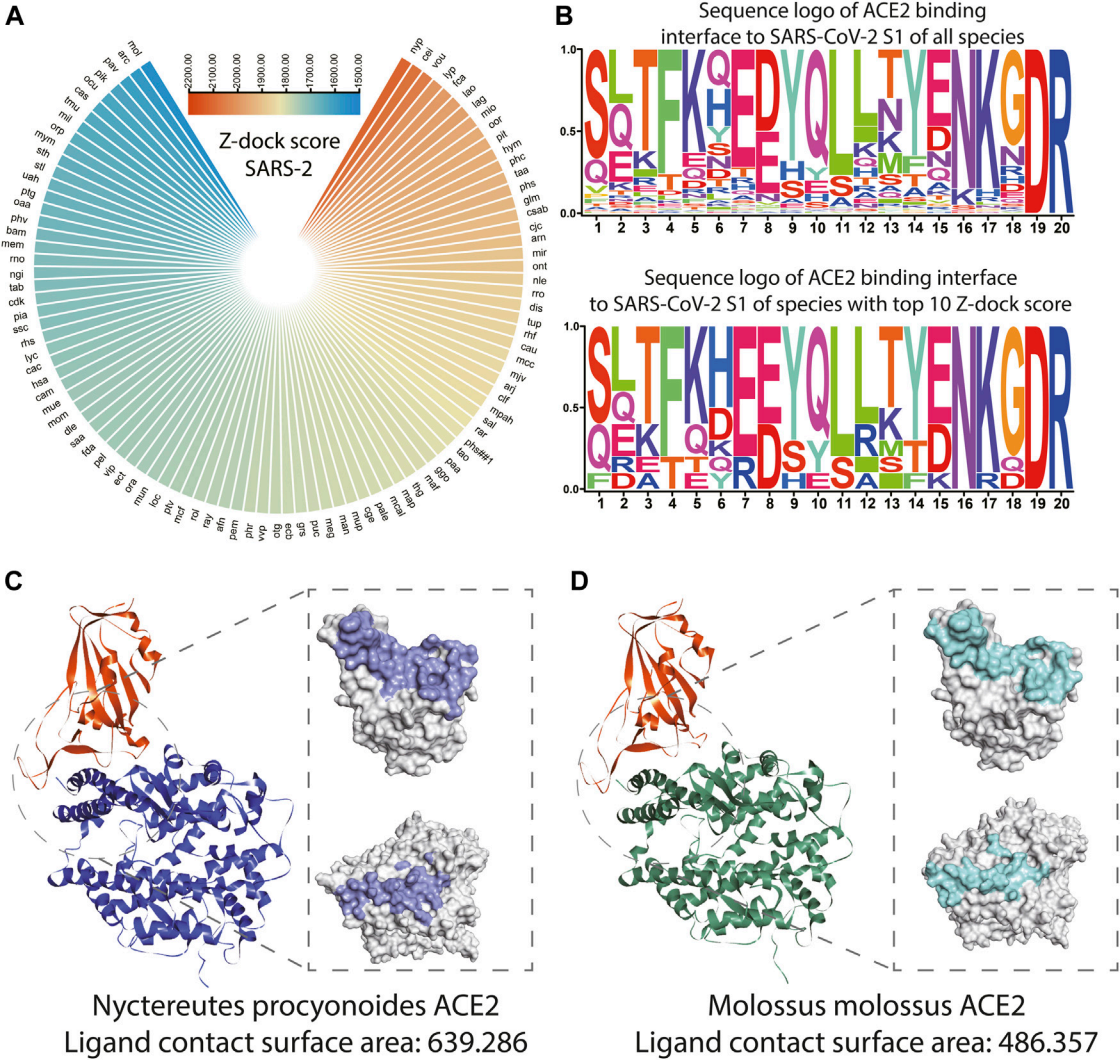
Structure and binding affinity analysis of SARS-CoV-2 S1 proteins with ACE2s among species. **(A)** Polarized heatmap showing the Z-dock score of SARS-CoV-2 S1 protein and host ACE2 among species. **(B)** Sequence logo of ACE2 binding interface to SARS-CoV-2 S1 of all species and sequence logo of ACE2 binding interface to SARS-CoV S1 of species with the top 10 Z-dock score. **(C)** Overall and contact surface structure of SARS-CoV-2 S1-RBD *in silico* docked with simulated ACE2 structure of *Nyctereutes procyonoides*. **(D)** Overall and contact surface structure of SARS-CoV-2 S1-RBD *in silico* docked with the simulated ACE2 structure of *Molossus molossus.*

As shown in Figure 4A and Supplementary Table S5, the top 10 species whose ACE2s are with the highest Z-dock score when docking with SARS-CoV-2 S1 were *Nyctereutes procyonoides*, *Cebus imitator*, *Vombatus ursinus*, *Lynx pardinus*, *Felis catus*, *Lagenorhynchus obliquidens*, *Lama glama*, *Microtus ochrogaster*, *Orcinus orca*, and *Piliocolobus tephrosceles*. For docking with SARS-CoV S1, species with the top 10 Z-dock score were *Tachyglossus aculeatus*, *Talpa occidentalis*, *Macaca fascicularis*, *Artibeus jamaicensis*, *Felis catus*, *Piliocolobus tephrosceles*, *Rhinopithecus roxellana*, *Meleagris gallopavo*, *Rattus rattus*, and *Rhinolophus ferrumequinum* (Supplementary Figure S4B).

The loci of the human binding site to S1 of SARS-CoV and SARS-CoV-2 (LBS) was extracted from the previous study (Shang et al., 2020), the un-gapped and unopened blast during homolog sequence extraction enabled direct extraction of corresponding loci from ACE2 of other species. The sequence logo was plotted with ACE2 LBS of SARS-CoV S1 and SARS-CoV-2 S1 of all species and species with the top 10 Z-dock score. It could be observed that ACE2 LBS to SARS-CoV-2 S1 of all the mammals involved in the analysis is conserved on the 19th and 20th amino acids, while the sixth, seventh, 13th, 15th, 16th, and 18th amino acids seem to be more conserved among the top 10 species. It should be noted that on the eighth loci, the most common amino acid is E, while D is the most common amino acid among all species (Figure 4B). In terms of ACE2 LBS to SARS-CoV S1, species with top 10 Z-dock scores showed higher conservatism on the 13th, 14th, and 15th amino acids. While it should be noted that all the mammals involved in the analysis are conserved on the 16th and 17th amino acids (Supplementary Figure S4C). The crystal structures and detailed interaction of ACE2 from *Nyctereutes procyonoides* or *Piliocolobus tephrosceles* and SARS-CoV-2 S1-RBD were analyzed by the Dock and Analyze Protein Complex Module of Discovery Studio. Consistent with the results of binding affinity analysis, the complex simulation of *Nyctereutes procyonoides* ACE2-RBD displayed a higher Z-dock score than *Piliocolobus tephrosceles* ACE2-RBD, which showed the highest and lowest Z-dock scores among the modeled structures. In addition, the contact surface area of SARS-CoV-2 S1-RBD in contact with ACE2 was calculated in our assays. We discovered that the contact surface area of *Nyctereutes procyonoides* ACE2 among the complex is 639.286, whereas that of SARS-CoV2 S1-RBD is 627.057 (Figure 4C). Notably, the area of *Piliocolobus tephrosceles* ACE2 in contact with S1-RBD is 486.357, and the interactive surface acre in RBD is 478.622 (Figure 4D). The larger interactive area between the complex simulation of *Nyctereutes procyonoides* ACE2-RBD explains why *Nyctereutes procyonoides* ACE2 displayed the highest Z-dock score when docked with SARS-CoV2 S1-RBD, rather than other species. Comparison of PDB: 7DF4 (SARS-CoV-2 S1 and human ACE2 complex) and simulated human ACE2 *in silico* docked with SARS-CoV-2 S1 was also conducted, which showed significant overlap on the structure (Figure S4d), indicating the reliability of our strategy of structuring modeling and *in silico* docking.

## Discussion

Detecting the general coronaviruses infection in the host can reduce the overall risk of infection and construct a coronavirus transmission network, which helps better prevention of coronavirus infection. In this study, we constructed a method, named *in silico* infection analysis (iSFA), which involves the transcriptome data mining from existing data available on the NCBI and could perform traceability and transmission risk evaluation of the coronaviruses and detect the coronavirus infection in various mammals including PEDV in *Equus burchellii.* Instead of detecting the DNA fragments imprinted in the genome, Souilmi et al. (2021) identified infection traces of coronaviruses in human East Asian populations with adaptive events in coronavirus-related genes, which needs datasets of population size. The iSFA strategy was to focus on more hosts such as wild mammals and detect their infected situation with less cost. By integrating the previous related studies with these iSFA results, a transmission network for several coronaviruses was constructed, which could provide an important resource for cross-species transmission risk evaluation. Mammals play a critical role in the diffusion of coronaviruses and contribute to the evolution of some kind of viruses (Costagliola et al., 2020), which made it significant to amplify the coronavirus transmission network. With the exponentially increasing number of deposited data from diverse tissues of vast species of animals, iSFA could be applied to detect virus genome fragments in multiple species to reveal the potential transmission route of a certain virus.

Rather than virus genome fragment detection, iSFA could be further utilized for expression analysis of the corresponding receptor that mediates the invasion and pathogenesis of the certain virus. In this study, receptor sequence similarity and expression patterns among 65 receptor genes and 26 non-human mammals were analyzed, respectively. Interestingly, PEDV infection was observed in *Sus scrofa* which can inhibit the expression of receptors of CoV_HKU15, SARS-CoV, and SARS-CoV-2 such as ANPEP, ACE2, CLEC4G, and CLEC4M. This result supported the hypothesis that the invasion of one virus may inhibit the invasion of another virus to ensure “the first invader” in an uncontested environment persist and proliferate (Hall et al., 1994), which may inhibit the expression of the receptor genes. With the rapidly increasing available amount of transcriptome data, especially the single-cell sequencing data from public databases, this analysis could shed more insights into the gene expression profile studies, such as the co-infection and co-expression of the receptor or co-receptor genes with a specific virus.

SARS-CoV and SARS-CoV-2 infection are initialized by the binding of S1 protein to the ACE2 receptor of the host. Thus, our *in silico* docking may benefit the construction of a landscape of the S1 binding to ACE2 of various species, which may reveal the infection risk of various mammals. It could be observed that *Nyctereutes procyonoides* (raccoon dog) ranks among the highest Z-dock score. And the detailed analysis for interactive surfaces among the modeled complexes also displayed a larger interface area on *Nyctereutes procyonoides* ACE2 and SARS-CoV2 S1-RBD, which agreed with the highest Z-dock score of complex simulation of *Nyctereutes procyonoides* ACE2-RBD. This consistency of contact area and the Z-dock score not only explained the variability of binding affinity among different species but also verified the reliability of our established *in silico* docking method. Previous studies also suggest the raccoon dog is the intermediate host of SARS-CoV and could also be infected by SARS-CoV-2 (Freuling et al., 2020). We also observed ACE2 of *Felis catus* (cat) showed a high Z-dock score when *in silico* docked with S1 of SARS-CoV and SARS-CoV-2, while the cat has also been reported as an intermediate host of SARS-CoV-2 (Stout et al., 2020). Reports of other species with high Z-dock scores infected with SARS-CoV-2 were failed to be found, but these species were geographically distributed distantly from human society, which might cause low chance of infection or their infection is rarely reported. Higher conservatism of LBS amino sequence could be observed in species with top 10 Z-dock scores, these loci might play an important role during ACE2 and S1 binding.

As for the conclusion, this study performed a comprehensive *in silico* analysis about the potential coronavirus mammalian host from different aspects and proposed an *in silico* infection analysis system that could be extensively applied to explore the viral infection in different species. The infection of PEDV in *Equus burchellii* was revealed by using this approach. Moreover, this *in silico* infection analysis suggested that the expression of coronavirus receptor genes tended to be downregulated after infection from another virus. Finally, we provided and verified a modeling and docking strategy, which revealed conserved loci of the SARS-CoV and SARS-CoV-2 S1 binding site of ACE2, and the higher conservatism of the LBS sequence in the species with top 10 Z-dock scores. Our iSFA including transcriptome data mining and *in silico* modeling and docking could suggest potential intermediate hosts of SARS-CoV-2 and other coronaviruses and could be extensively applied to investigate other virus infections in various species in the future. Though it still needs further verification through animal experiments and clinical studies, iSFA of transcriptome data mining and *in silico* modeling and docking could provide a reference to the methodology of the intermediate host of pathogen anticipation and epidemic control.

## Materials and Methods

### Transcriptomic Data

The mammalian classification information was obtained from The Catalogue of Life website. Transcriptome data of different mammalian from the lung and the intestine were obtained from the NCBI Sequence Read Archive (SRA), and those species including *Homo sapiens*, *Mus musculus*, and *Rattus norvegicus*, which have been frequently analyzed, were removed from further analysis. Also, those datasets from virus-infected experiments were removed for traceability analysis as well. After screening, 2,257 datasets were finally applied to further analyses, in which lung-associated 1,869 datasets belonged to 82 species (Supplementary Table S3) and intestine-associated 388 datasets belonged to 22 species (Supplementary Table S4).

### Complete Genome of Coronaviruses

Complete genomes of 38 coronaviruses were downloaded from the Virus Pathogen Resource. These coronaviruses derived from four genuses known as alpha-coronavirus, beta-coronavirus, delta-coronavirus, and gamma-coronavirus, which belong to Coronaviridae family and Orthocoronavirinae subfamily.

### Receptor Protein Sequences

A total of 24 respiratory virus receptors were collected from the literature, and the corresponding gene sequences and protein sequences were downloaded from the KEGG resource with R.

### Sequence Alignment

We took 38 coronaviruses except RaTG13 including AviCoV, Ro-Bat-CoV_HKU9, PEDV, HCoV-229E, HCoV-NL63, FIPV, Sc-BatCoV_512, SARS, MERS, SARS-CoV-2, BtCoV, BWCoV_SW1, BuCoV_HKU11, Camel-CoV_HKU23, Canada-goose_CoV, CmCoV_HKU21, AcCoV-JC34, CoV_HKU15 (PDCoV), EhCoV_HKU31, FCoV, Hedgehog_CoV, Hp-Bat_CoV_CHB25, HCoV_HKU1, Mi-Bat_CoV, Mink-CoV, MunCoV_HKU13, Murine-CoV, NHCoV_HKU19, PCoV_GX-P1E, RaTG13, Pi-BatCoV_HKU5, PEAV, QdCoV, Rh-Bat-CoV_HKU2, SpCoV, SeACoV, ThCoV_HKU12, Ty-BatCoV_HKU33, and Ty-BatCoV_HKU4 (Supplementary Table S1) into consideration.^17^ Because these data contains dozens of species, blastn (2.9.0+) (Shen et al., 2016) was chosen as aligner with complete virus genomes as reference. Those mapped reads whose E-value was less than 1e-5 and identity greater than 90% were counted for further downstream analysis. The virus infection traceability analysis also took genome coverage and unique viral genome fragments into consideration.

Multiple sequence alignments of spike glycoprotein nucleic acid sequences from 39 coronaviruses were displayed by MUSCLE (Edgar, 2004), while multiple sequence alignments of SARS-CoV-2 S1 protein fraction were carried out by ClustalW (Thompson et al., 2002) using sequences separated from animals including *Martes* (MT457393-MT457401), *Felinae* (MT457390-MT457392), *Panthera tigris* (MT365033), and *Canis lupus familiaris* (MT270814) released by GenBank, partial genomic sequence of the Guangdong pangolin coronavirus (GD/P1L) released by Lam et al. (2020), RaTG13 (MN996532.1) with genome highly similar to SARS-CoV-2 by GenBank and from *Homo sapiens* (MT676411.1) as reference.

### Expression Measurement of Coronavirus Receptors

The differences among these protein sequences were measured by alignment using the Needleman–Wunsch algorithm. Blastn was selected to aligned transcriptomic data into receptor reference sequences for expression evaluation. We took steps of quality control, which first considered sequencing depth and genome size, then retained the sequences of 26 mammalian species for further analysis with sequencing depth greater than 2 and genome C-value around 3 (Supplementary Figures S3A,B). Normalization of receptors expression referred to the standardized method of TPM to balance the impact of gene length, and sequencing depth and was performed as follows:

normalized expression=mapped reads numgene lengthΣ reads lengthgenome length.

Genome length was estimated by C-value downloaded from the Animal Genome Size Database and Σ reads length was statistics by seqkit (v0.12.1) (Shen et al., 2016).

### Receptor Expression Level Among Infected and Non-Infected Samples

Considering the sample size, we chose datasets from *Sus scrofa* to compare the expression of receptor genes between the infected and non-infected samples. We extracted non-infected and PEDV-infected expression data from intestines, and non-infected and PRRSV-infected data from the lung. Thereafter, the *t*-test was employed to examine whether virus infection will influence the expression of receptors significantly.

### Phylogenetic Tree and Cladogram Construction

Phylogenetic tree was generated by FastMe in a distance-based manner (Lefort et al., 2015) and using iTol^31^ for advance annotation and management, while the cladogram was generated using the same procedure but with the branch lengths ignored during the visualization.

### Protein Structure Simulation and Acquisition

The ACE2 protein sequences were acquired from the NCBI. The sequence and structure of ACE2 from the SARS-CoV S1–human ACE2 complex (PDB: 6acg) or the SARS-CoV-2 S1–human ACE2 complex (PDB: 7DF4) were extracted. The sequence was used as a template for the extraction of homolog sequence from the amino acid sequence of ACE2 of other species based on blast without gap and open. Extracted amino acid sequences were submitted to MODELLER (Webb and Sali, 2016) for the structure simulation with the extracted binding site structure mentioned earlier, while S1-RBD to ACE2 of SARS-CoV and SARS-CoV-2 was also extracted in the preparation of batch docking.

### Z-Dock

Z-Dock software was downloaded to run the batch docking locally. Ligand (S1-RBD) and receptor (ACE2 from different species) structures were submitted to Z-dock, and the score was extracted from each docking output file and wrote into a text file with name of their corresponding organism. The Z-dock score represents the binding affinity of each ligand and receptor complexes.

### Protein Docking and Complex Analysis

To understand the binding interaction of ACE2 receptors from different species with SARS-CoV-2 S1-RBD, the in-built protein docking module Z-dock in Discovery Studio 2018 software was employed to predict their binding poses. According to the protocol, first, the water molecules in receptors and ligands were removed before running the calculation to prevent the oxygen atom from being treated the same as an unknown atom in case if water is included. The proteins were prepared by Clean Protein tools to remove the alternative conformations, added the termini, and patched the missing side chain atoms. Thereafter, the 3D structures of ACE2 and SARS-CoV-2 S1-RBD were merged into a single Graphics View as a receptor and a ligand, respectively. The docking parameters were set as follows: angular step size was calculated with a defined value 15, RMSD cutoff was defined as 6.0, interface cutoff was defined as 9.0, maximum number of clusters was defined as 60, and other parameters were set as default. Finally, the docked protein complexes were optimized and re-scored using Refine Docked Protein tools. The docked protein complexes were analyzed and visualized by the Analyze Protein Interface. Polarized heatmaps indicating the binding affinity of S1 and ACE2, and the sequence logo of S1 binding site were both generated by TBtools (Chen et al., 2020a).

## Data Availability

The original contributions presented in the study are included in the article/Supplementary Material; further inquiries can be directed to the corresponding authors. The scripts for transcriptome data mining are available at https://github.com/GangCaoLab/iSFA. The scripts for protein modelling and in-silico docking are available at https://github.com/MSN-06s/Protein_modelling_and_docking.

## Supplementary Material

Supplementary Figure S1 Details of different coronaviruses infected situation in *Manis javanica*. **(A)**. Heatmap of sequence coverage on the viral genome. **(B)**. Heatmap of total hits that aligned to the coronaviral genome.

Supplementary Figure S2 Assembled genome of two coronaviruses. **(A)**. Coverage of fragments isolated from virus infected *Callithrix jacchus* where the blue blocks mean consistent with MERS-CoV genome. **(B)**. Coverage of fragments isolated from virus infected *Sus scrofa* where the blue blocks mean consistent with PEDV genome.

Supplementary Figure S3 Receptor gene expression analysis. **(A, B)**. Heatmap of natural logarithm of mean expression of 24 respiratory receptor genes in tissue intestine and lung of different species. **(C)**. Box plot of natural logarithm of 5 receptor genes expression level between PRRSV infected and non-infected group in lung of *sus scrofa*.

Supplementary Figure S4 Binding affinity analysis of SARS S1 proteins with ACE2s among species. **(A)**. Schema of protein structure simulation and *in-silico* docking. **(B)**. Polarized heatmap showing the z-dock score of SARS-CoV S1 protein and host ACE2 among species. **(C)**. Sequence logo of ACE2 binding interface to SARS-CoV S1 of all species and sequence logo of ACE2 binding interface to SARS-CoV S1 of species with top 10 Z-dock score. **(D)**. The overlay of simulated and docked human ACE2 + SARS-CoV-2 S1 structure on 7DF4.
