# Supplementary figures and images for: In Silico Infection Analysis (iSFA) Identified Coronavirus Infection and Potential Transmission Risk in Mammals
Source: Front Mol Biosci. 2022 Feb 8;9:831876. doi: 10.3389/fmolb.2022.831876 (PMC8861533; doi:10.3389/fmolb.2022.831876)

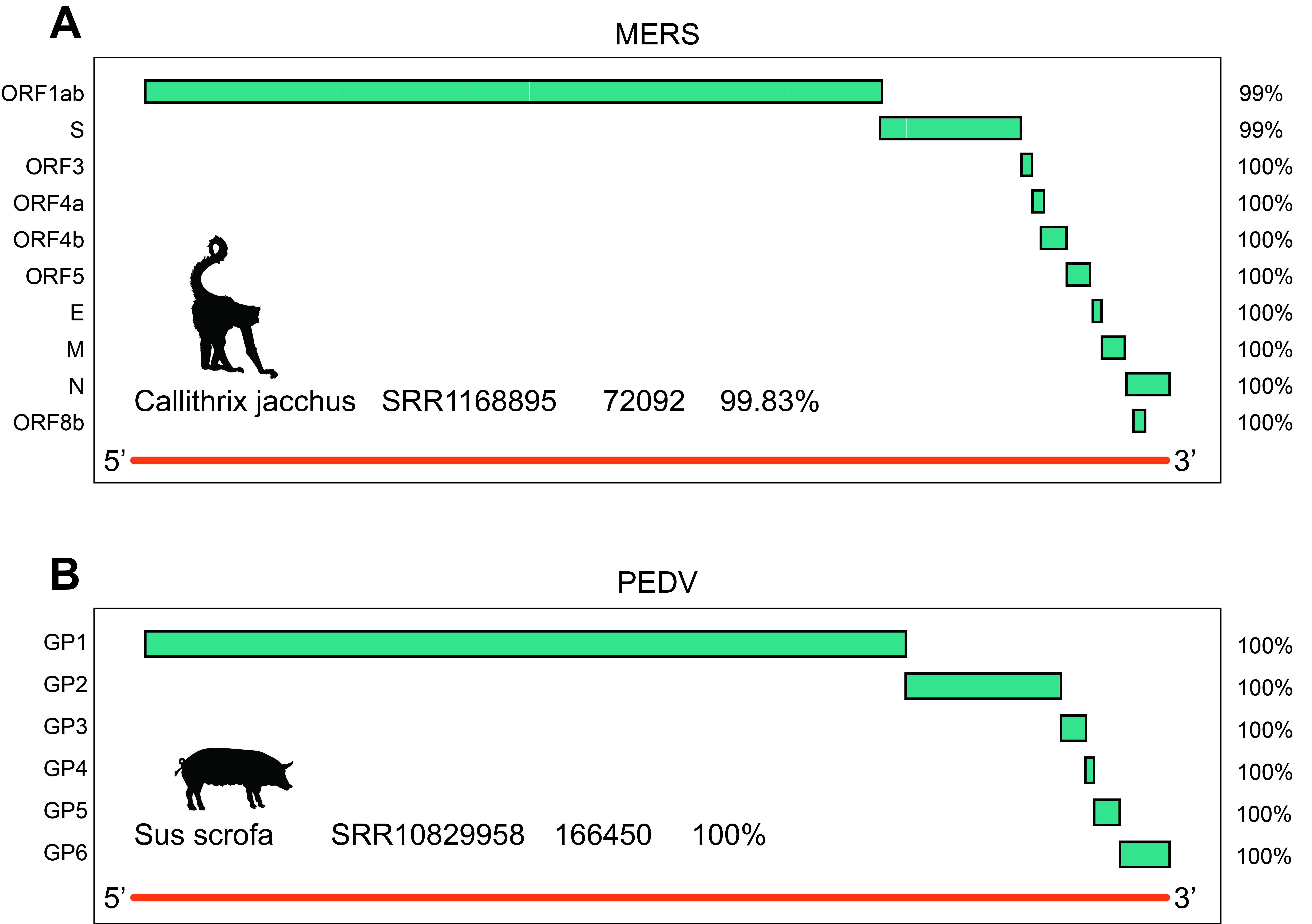

Supplement: Supplementary file 2 [file Image2.jpg]
